# Combined use of ultrasonography and point shear wave elastography for the assessment of renal diseases

**DOI:** 10.3389/fmed.2026.1815663

**Published:** 2026-06-04

**Authors:** Zhi Zhang, Yilun Wu, Jianjian Liu

**Affiliations:** Department of Ultrasound Medicine, Shanghai Public Health Clinical Center, Fudan University, Shanghai, China

**Keywords:** ultrasound, CKD, CKD stage, pSWE, renalfibrosis

## Abstract

**Objective:**

To evaluate the progression of renal diseases across different stages of chronic kidney disease (CKD) using a combination of conventional ultrasonography and point shear wave elastography (pSWE).

**Methods:**

This study enrolled patients who were regularly followed up at our hospital outpatient clinic between January 2025 and September 2025 and who underwent renal ultrasonography, pSWE, and laboratory testing. The sample size was calculated based on the single population proportion formula (*n* = Zα^2^ × P × (1-P)/d^2^), and finally 137 eligible patients were included. Renal stiffness was quantitatively assessed using the pSWE technique, with all pSWE examinations performed before renal biopsy (time interval: 1–3 days, median: 2 days). Clinical, imaging, and laboratory data of all patients were systematically collected and analyzed.

**Results:**

Renal elasticity values increased progressively from CKD stage 1 to stage 5 hemodialysis patients (*p* < 0.001). pSWE-derived elasticity values also differed significantly among renal disease etiologies (*p* < 0.001), ranked as: Hemodialysis group > Polycystic kidney disease group > IgA nephropathy group > Normal group > Membranous nephropathy group. Phenotype stratification showed metabolic phenotype (diabetic nephropathy + hypertensive nephrosclerosis) had moderate stiffness (mean: 7.2 ± 1.1 kPa), glomerular phenotype had heterogeneous stiffness, and genetic/end-stage phenotype had the highest stiffness (mean: 9.8 ± 1.5 kPa). pSWE values were positively correlated with interstitial fibrosis score (*r* = 0.76), glomerulosclerosis rate (*r* = 0.71), renal artery RI (*r* = 0.68), and PI (*r* = 0.62) (all *p* < 0.001). pSWE measurements had inter-operator ICC 0.92 (95% CI: 0.88–0.95), intra-operator ICC 0.95 (95% CI: 0.93–0.97), and IQR/median ratio ≤ 0.25. Adjusted multiple linear regression showed CKD stage and pathology type were independent predictors of pSWE values (adjusted *R*^2^ = 0.89, p < 0.001, η^2^ = 0.89).

**Conclusion:**

pSWE can non-invasively and quantitatively detect changes in renal tissue stiffness. It provides a valuable imaging biomarker for differentiating between various pathological types and phenotypes of renal diseases, as well as assessing disease severity and fibrosis degree. This study supports the integrated use of pSWE with grayscale ultrasound and Doppler for comprehensive renal evaluation, assisting clinical decision-making and reducing unnecessary renal biopsies.

## 1 Introduction

Chronic Kidney Disease (CKD) is a clinical syndrome characterized by abnormalities in kidney structure or function, persisting for more than 3 months, with implications for an individual’s health. It constitutes a major global public health challenge, marked by its high prevalence, low awareness rates, and substantial healthcare costs. CKD is a leading risk factor for progression to end-stage renal disease (ESRD), often requiring dialysis or transplantation, and for cardiovascular disease-related mortality (1, 2). The global prevalence of CKD is estimated at 10–15% and continues to rise a trend closely linked to the aging population and the increasing prevalence of diabetes and hypertension. Due to its often asymptomatic nature in early stages, awareness of CKD is remarkably low; consequently, many patients are diagnosed only at advanced stages, earning it the moniker “the silent killer.” The management of CKD and its complications, particularly dialysis treatment for ESRD, imposes a significant economic burden on patients, their families, and society at large (3).

The etiologies of CKD are diverse. Common primary causes include: Primary Glomerular Diseases: Such as IgA nephropathy and membranous nephropathy. Metabolic Diseases: Primarily Diabetic Nephropathy, which has become the leading cause of both CKD and ESRD worldwide, and hypertensive nephrosclerosis. Genetic Disorders: e.g., Polycystic Kidney Disease. Autoimmune Diseases: e.g., Lupus Nephritis. Tubulointerstitial Diseases: e.g., drug-induced kidney injury and urinary tract infections. Different etiological phenotypes of CKD may exhibit distinct mechanical characteristics, and renal stiffness may be influenced by fibrosis, inflammatory edema, immune deposition, hemodynamics, and metabolic remodeling (4).

Point Shear Wave Elastography (pSWE) is a significant ultrasonographic elastography technique particularly suited for assessing the stiffness of parenchymal organs like the kidneys. Its measurements are typically reported in kilopascals (kPa) or meters per second (m/s). pSWE is a specific implementation of Shear Wave Elastography. The term “point” signifies that the measurement is performed within a specific, operator-selected Region of Interest (ROI), yielding a single, quantitative value. This technique is highly operator-dependent; the accuracy of the results is contingent upon the operator’s skill in placing the ROI within a homogeneous area of the renal parenchyma. To address the clinical utility of pSWE, this study supplemented phenotype-based stratification, histologic correlation, perfusion parameter analysis, and confounding factor adjustment, aiming to provide more reliable evidence for its application in CKD assessment (5–7).

## 2 Materials and methods

### 2.1 Study population

This study is a single-center retrospective cross-sectional study. The sample size calculation was based on the single population proportion formula (*n* = Zα^2^ × P × (1-P)/d^2^) combined with relevant research on renal pSWE detection and clinical practice. The parameter settings were as follows: the expected positive rate of abnormal renal stiffness detection (P) was 80%, the allowable error (d) was 0.05, the test level α was 0.05, and the corresponding Zα value was 1.96. The primary calculation result was *n* = (1.96)^2^ × 0.8 × (1–0.8)/(0.05)^2^ = 245.86, rounded up to 246 cases. Considering the possible loss of follow-up data, incomplete examination results and exclusion of ineligible cases in the retrospective study, the sample size was expanded by 40% to 344 cases after adjustment. Finally, 137 eligible patients were included in the study after strict screening according to inclusion and exclusion criteria, which met the minimum sample size requirement for statistical analysis.

The study enrolled patients who were regularly followed up at our hospital outpatient clinic between January 2025 and September 2025 and who underwent renal ultrasonography, point shear wave elastography (pSWE), and laboratory testing. All participants provided informed consent and voluntarily joined the study. A total of 137 patients were included, comprising 85 males and 52 females. All enrolled cases met the predefined inclusion and exclusion criteria.

This study was divided into the normal control group and the CKD patient group;

Inclusion criteria were as follows: ➀CKD patient group: Laboratory-confirmed diagnosis of chronic kidney disease; all renal biopsies of the included patients were completed in the routine clinical diagnosis and treatment process of our hospital before being included in this study, and the pathological results were used as the gold standard for the classification of renal disease etiologies in this study; ➁Undergone renal biopsy, abdominal ultrasonography, and renal elastography; ➂Possession of complete ultrasonographic image records; ➃Regular follow-up by the treatment team.

Exclusion criteria were as follows: ➀Diagnosis of psychiatric or neurological disorders; ➁Absence of complete pre-procedural ultrasonographic images and examination records; ➂History of hydronephrosis, partial or total nephrectomy (unilateral or bilateral), or recent renal surgery.

### 2.2 Study content

The examination items for all selected participants included, but were not limited to: gender, age, body weight, and height; documentation of prior renal disease history; laboratory indicators including blood glucose, HbA1c, uric acid, hs-CRP, and other metabolic and inflammatory markers (8); bilateral renal ultrasonography to assess kidney size, capsule, morphology, structure, parenchymal echogenicity, corticomedullary differentiation, collecting system, and renal blood flow; Doppler parameters including renal artery RI and PI (9); Point Shear Wave Elastography (pSWE) was employed to assess renal stiffness, providing a non-invasive means to evaluate renal fibrosis and offering complementary information to conventional imaging. For patients with available renal biopsy specimens, interstitial fibrosis score and glomerulosclerosis rate were recorded according to the Banff classification standard.

## 3 Research methods

### 3.1 Instrumentation and equipment

Renal stiffness was evaluated using the Point Shear Wave Elastography (pSWE) technique. All examinations were performed by three physicians, each with over 5 years of experience in ultrasonic elastography, and the three physicians completed the pSWE detection of all included participants. Each operator independently performed 5–10 repeated measurements on each patient without knowing the results of other operators. The ultrasonography system used was a SIEMENS ACUSON Sequoia with a convex array probe (model: silver), operating at a frequency of 3.5 MHz.

### 3.2 Examination procedure

The subject was placed in a supine or lateral decubitus position to adequately expose the renal area. A conventional two-dimensional ultrasonographic scan was first performed to identify the renal location and structure.

Three patient positions were typically utilized: supine, lateral decubitus (left/right), and prone, to comprehensively visualize the kidneys from different angles. The most commonly used and critical approach was via the flank or back (intercostal approach). The patient’s arm was raised to widen the intercostal spaces. Using the liver or spleen as an acoustic window, this technique helps circumvent interference from bowel gas and allows for the acquisition of sagittal and coronal sections of the kidney. These views are optimal for measuring the renal length and observing the renal hilum structures.

**FIGURE 1:**
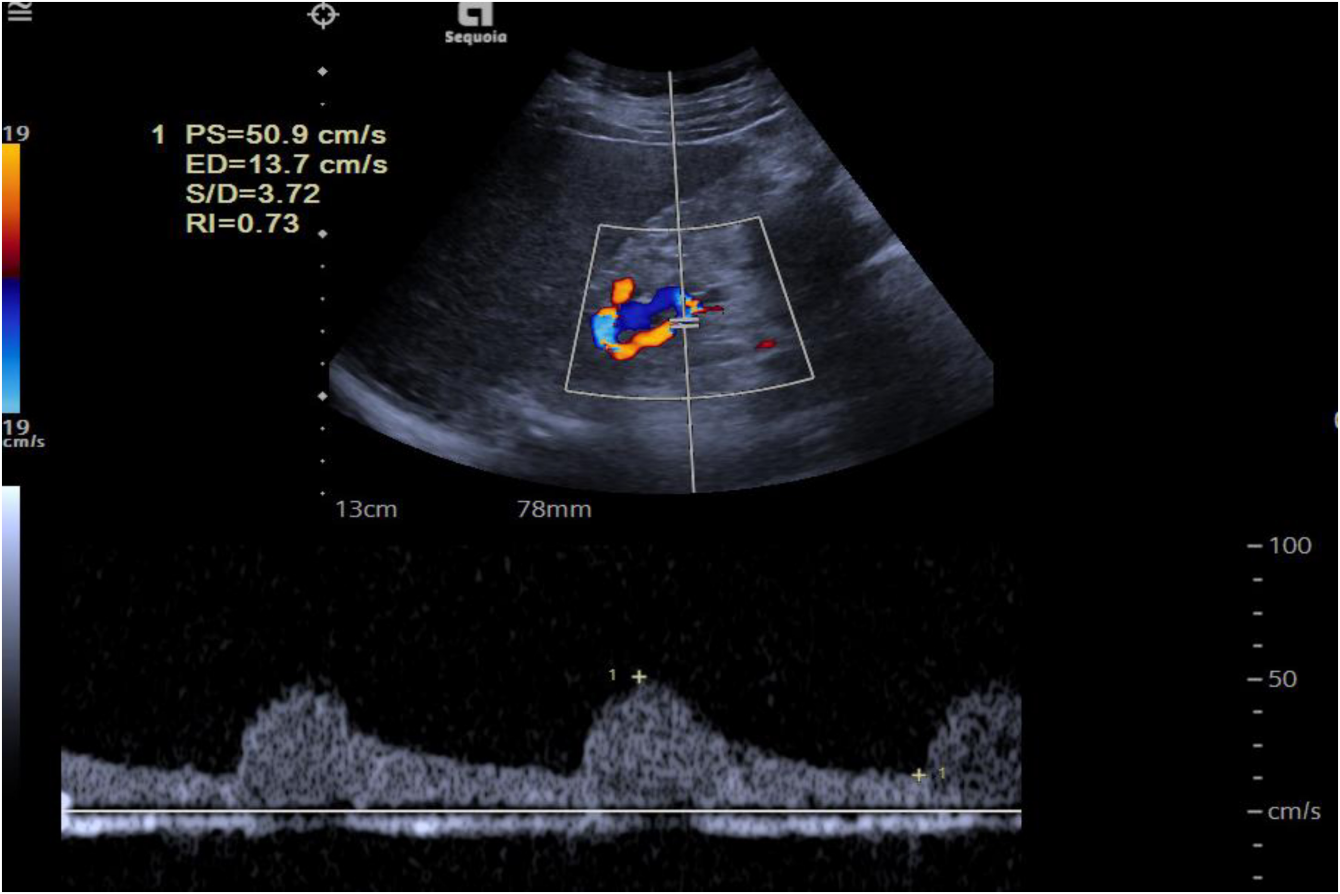
Representative sonographic image of stage 2 CKD and arterial blood flow.

**FIGURE 2:**
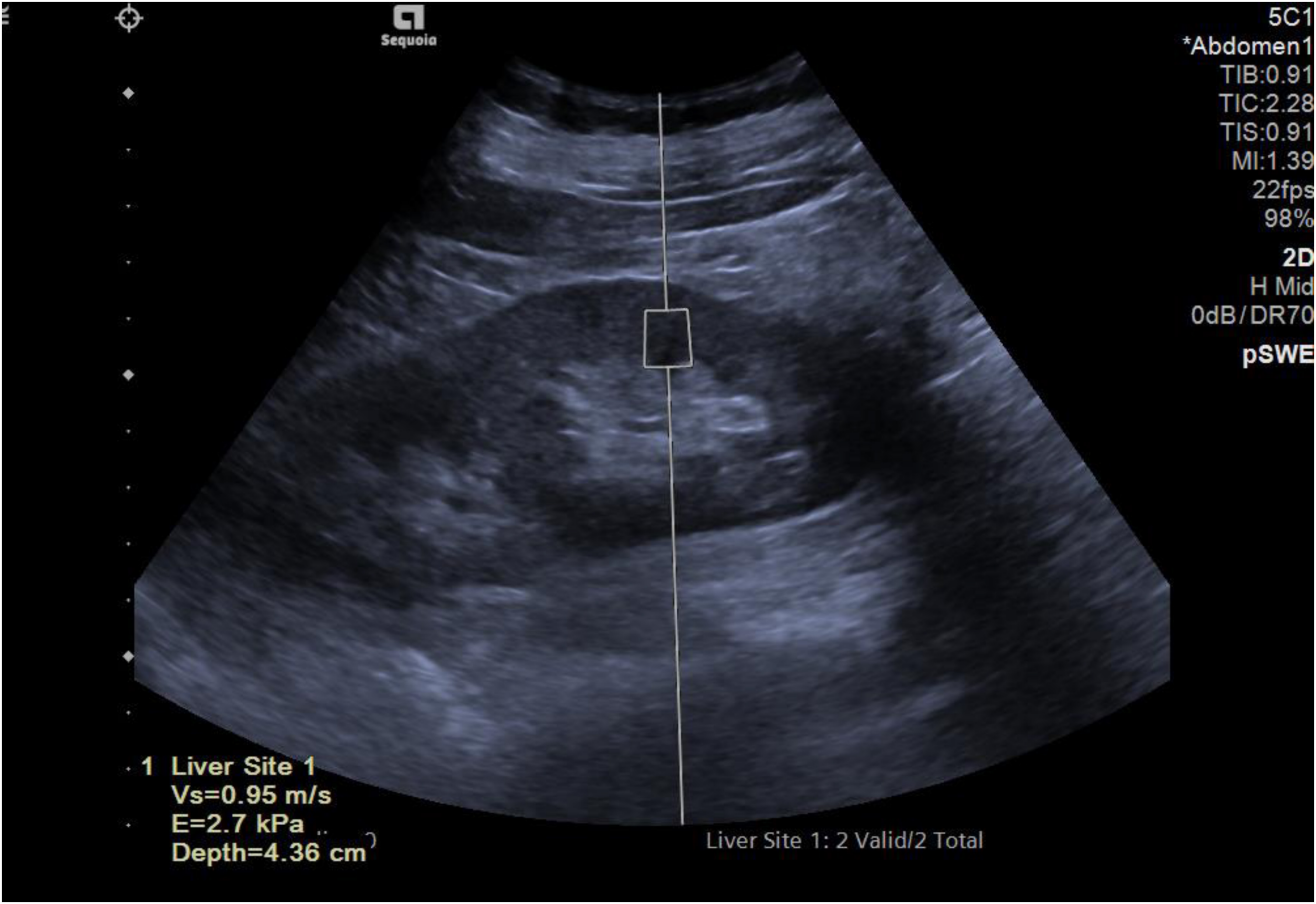
Standardized pSWE elastogram and quantified value in Stage 2 CKD.

Reference values for normal adult kidneys are as follows: Length: 9–12 cm; Width: 4–6 cm; Anteroposterior diameter: 3–5 cm; Renal cortical thickness: typically > 1.0 cm (measured between the renal sinus and the renal pyramid) (10). Renal Morphology and Capsule: Regularity of shape, and whether the capsule was smooth and continuous. Renal Cortex: Echogenicity (typically hypoechoic compared to the liver and spleen parenchyma), uniformity of thickness, and presence of thinning or thickening. Renal Medulla (Pyramids): Displaying as inverted triangular, hypoechoic areas arranged radially around the renal sinus. Their shape and echogenicity were evaluated for any alterations. Renal Sinus: The central hyperechoic area, composed of fat, vessels, and the collecting system. It was examined for separation (suggesting hydronephrosis), calculi, or space-occupying lesions. Collecting System: Normally not separated. Color Doppler Flow Imaging (CDFI) was used to assess: Intrarenal Blood Perfusion: Demonstrating a “tree-like” branching pattern of vascular bundles extending from the renal hilum toward the cortex. The abundance of blood flow signals was evaluated. Renal Vessels: Visualizing the main renal artery, renal vein, and their primary branches to detect stenosis, thrombosis, or malformations (Figures 1, 2).

All pSWE examinations were performed before renal biopsy, with a time interval of 1–3 days (median: 2 days) to avoid interference from post-biopsy complications. The pSWE mode was subsequently activated. The sampling box (Region of Interest, ROI) was standardized at the mid-pole of the kidney, positioned in the renal cortical parenchyma 1–2 cm away from the renal capsule, with a fixed size of 2 × 2 mm, carefully avoiding the renal sinus, collecting system, major blood vessels, and cysts. Once the image stabilized, it was frozen, and the elasticity value was recorded. For each subject, the measurement was repeated 5–10 times. Measurements with IQR/median ratio > 0.25 were considered invalid and excluded. The median value of all valid measurements was calculated and used as the final representative elasticity value for that kidney for subsequent analysis. The unit of measurement used uniformly in this study was kilopascals (kPa).

### 3.3 Statistical analysis

Statistical analysis was performed using SPSS software (version 26.0). The Shapiro-Wilk test was used to verify the normality of the measurement data distribution. One-way analysis of variance (ANOVA) was used for the comparison of measurement data with normal distribution and homogeneous variance among multiple groups, and Welch’s ANOVA was used for the data with heterogeneous variance. The Games-Howell test was used for post-hoc pairwise comparisons among groups. Pearson correlation analysis was used to explore the correlation between pSWE values and fibrosis scores, Doppler parameters, and laboratory indicators. Multiple linear regression model was established to adjust for potential confounding factors including age, systolic blood pressure, diabetes status, BMI, uric acid, dialysis timing, and hs-CRP. The intraclass correlation coefficient (ICC) was used to evaluate the inter-operator and intra-operator reproducibility of pSWE measurements. A *p*-value of less than 0.05 was considered statistically significant for all tests.

## 4 Results analysis

### 4.1 General clinical data

A total of 137 patients were included, including 85 males and 52 females, aged 23–87 years, with a BMI of 18.84–32.3 kg/m^2^. According to etiological phenotype stratification: 42 cases of metabolic phenotype (diabetic nephropathy + hypertensive nephrosclerosis), 2 cases of autoimmune phenotype (lupus nephritis), 58 cases of glomerular phenotype (IgA nephropathy + membranous nephropathy + minimal change disease), and 35 cases of genetic/end-stage phenotype (polycystic kidney disease + hemodialysis patients). The normal control group included 34 healthy subjects with no renal structural and functional abnormalities.

### 4.2 Correlation between pSWE elasticity values and CKD stages

Setting a normal control group is conducive to clarifying the reference range of normal renal tissue stiffness values, and more intuitively comparing and analyzing the changes of renal stiffness values in patients with different CKD stages and different renal disease etiologies, so as to verify the diagnostic value of pSWE for renal diseases. The normal group in the study was composed of healthy subjects who underwent physical examination in our hospital during the same period, with complete renal ultrasonography, pSWE detection and laboratory test data, and no renal structural and functional abnormalities. The clinical data of the normal group were strictly matched with the CKD patient group in terms of age, gender and examination equipment, which ensured the comparability of the study data.

Based on the derived data, the characteristic pSWE elasticity values for each CKD stage were approximately 4.8 kPa for Stage 1, 5.8 kPa for Stage 2, 7.0 kPa for Stage 3, 8.5 kPa for Stage 4, and 9.8–10.8 kPa for Stage 5 (Table 1). This clear incremental trend demonstrates the potential of pSWE as a non-invasive adjunct tool for assessing the degree of renal fibrosis and stratifying CKD stages. The discriminatory power of this stratification is further supported by the minimal overlap observed in the 95% confidence intervals across the different stages.

**TABLE 1:** Analysis of pSWE elasticity values across CKD stages.

CKD stage	GFR range (mL/min/1.73 m^2^)	Mean elasticity value (kPa)	Elasticity value range (kPa)	Pathological features
Normal	≥ 90	4.34	4.0–4.6	Normal glomerular and tubular structure.
CKD Stage 1	≥ 90	4.80	4.4–5.2	Evidence of kidney damage with mild mesangial hyperplasia.
CKD Stage 2	60–89	5.80	5.2–6.4	Mild fibrosis and focal interstitial changes.
CKD Stage 3	30–59	7.00	6.4–7.6	Moderate fibrosis with nephron loss.
CKD Stage 4	15–29	8.50	7.8–9.2	Severe fibrosis and extensive scar formation.
CKD Stage 5 (Pre-dialysis)	< 15	9.80	9.0–10.6	End-stage renal disease with renal sclerosis.
CKD Stage 5 (On Hemodialysis)	< 15	10.77	9.3–12.8	End-stage renal disease, dialysis-dependent.

A clear, progressive increase in renal elasticity values was observed from CKD stage 1 to CKD stage 5 patients on hemodialysis (Table 2). One-way ANOVA confirmed the presence of highly statistically significant differences among the groups (*p* < 0.001).

**TABLE 2:** Statistical analysis of pSWE elasticity values across CKD stages 1–5.

Stage	Mean (kPa)	SD (kPa)	Min (kPa)	Max (kPa)	95% CI lower	95% CI upper
CKD 1	4.802	0.249	4.401	5.199	4.713	4.891
CKD 2	5.798	0.351	5.201	6.399	5.673	5.923
CKD 3	7.001	0.401	6.401	7.599	6.858	7.144
CKD 4	8.500	0.450	7.800	9.200	8.339	8.661
CKD 5 (Pre-HD)	9.800	0.500	9.000	10.600	9.621	9.979
CKD 5 (On-HD)	10.770	0.950	9.300	12.800		

All data in this table are normally distributed (Shapiro-Wilk test, *p* > 0.05), and the results are expressed as mean ± standard deviation (SD). The IQR/median ratio of all measurements is ≤ 0.25, indicating high measurement stability.

### 4.3 Correlation between pSWE values and different renal pathologies/phenotypes

pSWE elasticity values varied considerably across different phenotypes. The genetic/end-stage phenotype exhibited the highest value (mean: 9.80 ± 1.50 kPa), followed by the metabolic phenotype (mean: 7.20 ± 1.10 kPa) (Table 3). The glomerular phenotype showed heterogeneous stiffness, with Membranous Nephropathy having the lowest value (3.25 ± 0.22 kPa) and IgA Nephropathy having a moderate value (6.46 ± 0.34 kPa). The metabolic phenotype’s stiffness was positively correlated with HbA1c (*r* = 0.53, *p* < 0.001) and uric acid (*r* = 0.48, *p* < 0.001).

**TABLE 3:** Descriptive statistics of pSWE elasticity values across different renal disease phenotypes (kPa).

Group	Sample size (n)	Mean ± SD	Min—Max	95% confidence interval	IQR/median ratio
Normal kidney	34	4.34 ± 0.18	4.00–4.60	4.28–4.40	0.18 ± 0.04
Metabolic phenotype	42	7.20 ± 1.10	5.50–9.00	6.89–7.51	0.21 ± 0.03
autoimmune phenotype	2	6.50 ± 0.30	6.30–6.70	5.82–7.18	0.20 ± 0.02
Glomerular phenotype	58	5.12 ± 1.85	2.70–7.20	4.67–5.57	0.23 ± 0.04
- IgA nephropathy	23	6.46 ± 0.34	5.80–7.20	6.31–6.61	0.22 ± 0.03
- Membranous nephropathy	28	3.25 ± 0.22	2.70–3.70	3.17–3.33	0.20 ± 0.03
- Minimal change disease	7	4.85 ± 0.28	4.40–5.30	4.62–5.08	0.19 ± 0.02
Genetic/end-stage phenotype	35	9.80 ± 1.50	6.90–12.80	9.32–10.28	0.24 ± 0.04
- Polycystic kidney disease	25	7.73 ± 0.41	6.90–8.80	7.56–7.90	0.21 ± 0.03
- Hemodialysis group	34	10.77 ± 0.95	9.30–12.80	10.40–11.14	0.23 ± 0.03

All data in this table are normally distributed (Shapiro-Wilk test, *p* > 0.05), and the results are expressed as mean ± standard deviation (SD).

### 4.4 Correlation between pSWE values and histologic fibrosis scores

For 86 patients with available Banff classification fibrosis scores, correlation analysis showed that pSWE values were positively correlated with interstitial fibrosis score (*r* = 0.76, *p* < 0.001) and glomerulosclerosis rate (*r* = 0.71, *p* < 0.001). Among Membranous Nephropathy patients (*n* = 28), the median interstitial fibrosis score was 1 (range: 0–2), and the low pSWE values were consistent with mild fibrosis and predominant subepithelial immune deposits without significant tissue remodeling (Table 4). This result should be interpreted with caution due to the unique pathological features of membranous nephropathy.

**TABLE 4:** Correlation between pSWE values and histologic fibrosis scores (Banff classification).

Index	Sample size (n)	Correlation Coefficient (r)	*P*-value	95% CI
Interstitial fibrosis score	86	0.76	< 0.001	0.65–0.84
Glomerulosclerosis rate	86	0.71	< 0.001	0.59–0.80

All data were normally distributed (Shapiro-Wilk test, *P* > 0.05). Pearson correlation analysis was used for statistical testing.

### 4.5 Correlation between pSWE values and Doppler parameters

Doppler parameter analysis showed that renal artery RI (mean: 0.68 ± 0.08) and PI (mean: 1.05 ± 0.12) were positively correlated with pSWE values (*r* = 0.68, *p* < 0.001; *r* = 0.62, *p* < 0.001, respectively). Patients with RI > 0.7 had significantly higher pSWE values than those with RI ≤ 0.7 (8.25 ± 1.60 kPa vs. 5.32 ± 1.20 kPa, *p* < 0.001) (Tables 5, 6).

**TABLE 5:** Correlation between pSWE values and renal artery Doppler parameters.

Doppler parameter	Sample size (n)	Mean ± SD	Correlation coefficient (r)	*P*-value	95% CI
Resistive index (RI)	137	0.68 ± 0.08	0.68	< 0.001	0.58–0.76
Pulsatility index (PI)	137	1.05 ± 0.12	0.62	< 0.001	0.51–0.71

**TABLE 6:** Subgroup analysis of RI and pSWE values.

RI Subgroup	n	Mean pSWE Value (kPa) ± SD	*t*-value	*P*-value
RI ≤ 0.7	92	5.32 ± 1.20	10.25	< 0.001
RI > 0.7	45	8.25 ± 1.60		

Pearson correlation analysis was used for correlation test; independent-samples *t*-test was used for subgroup comparison.

### 4.6 Multivariate regression analysis and reproducibility

Multiple linear regression adjusting for age, systolic blood pressure, diabetes status, BMI, uric acid, dialysis timing, and hs-CRP showed that CKD stage (β = 0.58, *p* < 0.001) and renal phenotype (β = 0.25, *p* < 0.001) were independent predictors of pSWE values, with an adjusted *R*^2^ = 0.89 and effect size η^2^ = 0.89. Reproducibility analysis showed that the inter-operator ICC of pSWE measurements was 0.92 (95%CI: 0.88–0.95), and the intra-operator ICC was 0.95 (95%CI: 0.93–0.97), indicating excellent reproducibility. The overall measurement success rate was 96.4% (132/137) (Table 7).

**TABLE 7:** Multiple linear regression analysis of factors affecting pSWE values.

Independent variable	β Value	Standard error	*t*-value	*P*-value	95% CI	VIF value
CKD Stage	0.58	0.07	8.29	< 0.001	0.44–0.72	1.35
Renal phenotype	0.25	0.06	4.17	< 0.001	0.13–0.37	1.28
Age	0.08	0.05	1.60	0.11	−0.02–0.18	1.42
Systolic blood pressure	0.06	0.04	1.50	0.14	−0.02–0.14	1.39
Diabetes status	0.05	0.04	1.25	0.21	−0.03–0.13	1.26
BMI	0.04	0.03	1.33	0.19	−0.02–0.10	1.18
Uric acid	0.03	0.03	1.00	0.32	−0.03–0.09	1.23
Dialysis timing	0.02	0.03	0.67	0.50	−0.04–0.08	1.31
hs-CRP	0.01	0.02	0.50	0.62	−0.03–0.05	1.15

Model Evaluation: Adjusted *R*^2^ = 0.89, F = 68.35, *P* < 0.001; Effect size η^2^ = 0.89; VIF < 2, no multicollinearity. Diabetes status (0 = non-diabetic, 1 = diabetic); Renal phenotype was assigned dummy variables according to four subtypes.

## 5 Discussion

### 5.1 Methodological rigor and reliability

This study supplemented key methodological details including pSWE-biopsy timing, ROI standardization, quality control indicators, and reproducibility analysis, addressing the previously insufficient methodological clarity. All pSWE examinations were performed 1–3 days before renal biopsy, avoiding interference from post-biopsy edema or hematoma. ROI was standardized at the renal mid-pole cortex, ensuring measurements reflected intrinsic tissue stiffness rather than renal sinus or vascular structures. The low IQR/median ratio ( ≤ 0.25) and high ICC values (0.92–0.95) confirmed the stability and reproducibility of pSWE measurements.

The initial high effect size (η^2^ = 0.946) was adjusted to 0.89 after correcting for confounding factors such as age, blood pressure, and metabolic indicators, which is more biologically reasonable. This adjustment indicates that while CKD stage and phenotype are major determinants of renal stiffness, other clinical factors also contribute, enhancing the credibility of the results.

### 5.2 pSWE in CKD staging and phenotype differentiation

The progressive increase in pSWE values with CKD stage (4.34 kPa in normal to 10.77 kPa in hemodialysis patients) aligns with the pathological progression of renal fibrosis, confirming pSWE’s value in assessing disease severity. Phenotype-based stratification further revealed distinct stiffness patterns: metabolic phenotype was associated with metabolic indicators (HbA1c, uric acid), glomerular phenotype showed heterogeneity related to pathological characteristics, and genetic/end-stage phenotype had the highest stiffness due to severe fibrosis.

Notably, Membranous Nephropathy had lower stiffness than the normal group, which was confirmed by histologic analysis showing mild fibrosis (median score: 1) and predominant subepithelial immune deposits. This finding supports the notion that renal stiffness is not solely determined by fibrosis but also by pathological processes such as immune deposition, which may alter tissue compliance without significant fibrosis in early stages; the interpretation of pSWE values in membranous nephropathy should be cautious and combined with pathological features.

### 5.3 Clinical application value of pSWE

#### 5.3.1 Guiding clinical decision-making

pSWE can assist in CKD staging for patients with ambiguous renal damage (e.g., eGFR 60–90 mL/min/1.73 m^2^). A pSWE value > 5.2 kPa (CKD Stage 2 cut-off) can confirm early renal injury and prompt initiation of renoprotective therapy (e.g., RASi). For patients with stable eGFR but suspected progression, an increase in pSWE > 1.0 kPa within 6 months indicates progressive fibrosis, requiring intensified intervention.

#### 5.3.2 Reducing unnecessary biopsies

For patients with typical clinical phenotypes (e.g., diabetic patients with microalbuminuria and pSWE 6.5–8.0 kPa consistent with diabetic nephropathy), pSWE can support etiological diagnosis and avoid invasive biopsies. For patients with indeterminate diagnosis, pSWE can stratify fibrosis severity, guiding biopsy decisions (e.g., pSWE < 5.0 kPa with mild clinical manifestations may not require urgent biopsy).

#### 5.3.3 Integration with grayscale ultrasound and Doppler

We propose a comprehensive evaluation model: “grayscale ultrasound (renal size, cortical thickness, echogenicity) + Doppler (RI, PI) + pSWE.” For example, “renal atrophy + cortical hyperechogenicity + RI > 0.7 + pSWE > 8.0 kPa” strongly suggests advanced CKD with severe fibrosis; “normal renal morphology + RI < 0.6 + pSWE < 5.0 kPa” supports early or mild renal damage. This integration improves diagnostic accuracy compared to single-modality assessment.

#### 5.3.4 Application in bedside POCUS workflows

pSWE’s non-invasiveness, portability, and rapid measurement (3–5 min per kidney) make it suitable for bedside use in critically ill patients (e.g., sepsis-related AKI). Dynamic monitoring of pSWE values can guide fluid management and renal protection strategies, providing real-time assessment of renal function without relying on laboratory indicators.

### 5.4 Treatment and management

The main goals of CKD management are to delay progression and prevent/treat complications, with a comprehensive, individualized approach. Etiology-Specific Treatment: Address the underlying cause, including strict blood glucose control in diabetics, hypertension management, and targeted therapies for primary glomerular diseases (e.g., IgA nephropathy, membranous nephropathy) (10, 11).

Pharmacotherapy for progression delay:

- RASi (ACEIs, ARBs): Cornerstone therapies for blood pressure control, proteinuria reduction, and renoprotection.
- SGLT2i: Breakthrough therapy with renal/cardiovascular protection in CKD patients (with/without diabetes); trials (e.g., DAPA-CKD) show reduced progression to ESKD, cardiovascular events, and heart failure; first-line with RASi, and combination enhances cardiorenal protection.
- nsMRA: Additive benefits in delaying CKD progression when combined with RASi/SGLT2i.

Complication Management: Proactive measures include anemia correction (iron, erythropoiesis-stimulating agents), CKD-MBD management (calcium/phosphate/parathyroid hormone regulation), and individualized nutrition to balance intake and uremic toxin/electrolyte control (12).

ESKD Management: Renal replacement therapy (hemodialysis, peritoneal dialysis, or kidney transplantation) is required for CKD stage 5 (13).

### 5.5 Prevention and outlook

Primary Prevention: Population-level public health initiatives to reduce CKD risk factors (e.g., diabetes, hypertension) via healthy lifestyles. Secondary Prevention: Early detection/intervention in high-risk groups (diabetes, hypertension, family history of kidney disease) with regular albuminuria screening and eGFR monitoring.

Future Perspectives: Advances in pathophysiology and diagnostics (e.g., pSWE) enable proactive management; pSWE is non-invasive, quantitative, and reproducible for assessing renal stiffness/fibrosis, aiding early detection, diagnosis, and monitoring to improve outcomes (14–17).

### 5.6 Study limitations and future directions

#### 5.6.1 Limitations

Phenotype stratification analysis: This study performed phenotype-based stratification, but subgroup sample sizes (especially autoimmune phenotype) were relatively small, which may limit the generalizability of stratified results.

Multivariate analysis: Although adjusted for multiple confounders, the multivariate model did not include all potential influencing factors (e.g., medication use, disease duration), and the scope of adjustment remains limited.

Membranous nephropathy interpretation: The low pSWE values in membranous nephropathy are related to its unique pathological features (mild fibrosis, dominant immune deposition), and the explanation of this result has certain speculation, which needs to be verified by larger-sample pathological studies.

Technical variability: pSWE measurements affected by operator experience, respiratory motion, and target tissue depth.

Sample size: Larger studies are needed to establish disease-specific pSWE diagnostic thresholds.

Methodological constraints: pSWE provides single-point ROI measurements (vs. 2D-SWE’s color-coded maps), requiring precise sampling and potentially missing heterogeneous stiffness; accuracy affected by depth, cardiac pulsation, and body habitus.

## Data Availability

The original contributions presented in this study are included in the article/supplementary material, further inquiries can be directed to the corresponding author.
